# Cenpj/CPAP regulates progenitor divisions and neuronal migration in the cerebral cortex downstream of Ascl1

**DOI:** 10.1038/ncomms7474

**Published:** 2015-03-10

**Authors:** Patricia P. Garcez, Javier Diaz-Alonso, Ivan Crespo-Enriquez, Diogo Castro, Donald Bell, François Guillemot

**Affiliations:** 1 Division of Molecular Neurobiology, MRC National Institute for Medical Research, Mill Hill, London NW7 1AA, UK; 2 Department of Biochemistry and Molecular Biology I, School of Biology and Instituto Universitario de Investigaciones Neuroquímicas (IUIN), Complutense University, 28040 Madrid, Spain; 3 Department of Craniofacial Development & Stem Cell Biology, King’s College London, Guy’s Tower Wing, Floor 27, London SE1 9RT, UK; 4 Confocal and Image Analysis Laboratory, MRC National Institute for Medical Research, Mill Hill, London NW7 1AA, UK

## Abstract

The proneural factor Ascl1 controls multiple steps of neurogenesis in the embryonic brain, including progenitor division and neuronal migration. Here we show that *Cenpj*, also known as *CPAP*, a microcephaly gene, is a transcriptional target of Ascl1 in the embryonic cerebral cortex. We have characterized the role of *Cenpj* during cortical development by *in utero* electroporation knockdown and found that silencing *Cenpj* in the ventricular zone disrupts centrosome biogenesis and randomizes the cleavage plane orientation of radial glia progenitors. Moreover, we show that downregulation of *Cenpj* in post-mitotic neurons increases stable microtubules and leads to slower neuronal migration, abnormal centrosome position and aberrant neuronal morphology. Moreover, rescue experiments shows that *Cenpj* mediates the role of Ascl1 in centrosome biogenesis in progenitor cells and in microtubule dynamics in migrating neurons. These data provide insights into genetic pathways controlling cortical development and primary microcephaly observed in humans with mutations in *Cenpj.*

## Abstract (web summary)

The proneural factor Ascl1/Mash1 is an important regulator of embryonic neurogenesis. Here the authors identify that the microcephaly protein Cenpj/CPAP is essential for several microtubule-dependent steps in the neurogenic program driven by Ascl1 in the developing cerebral cortex.

## Graphical abstract

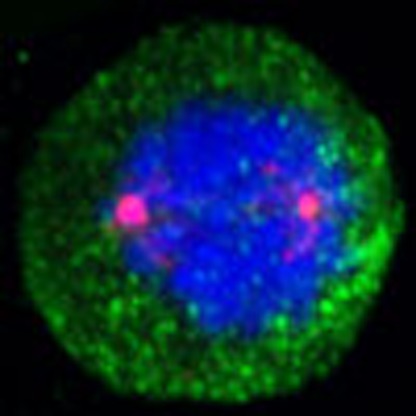

For the cerebral cortex to exert its crucial functions in cognition in humans, the different stages of its development, including the proliferation of its progenitors and the migration of its neurons, must proceed unencumbered. Defects in any of those steps can result in severe disorders, including microcephaly, lissencephaly or microlissencephaly^1^,^2^. Investigation of the mechanisms that control the neurogenesis thus contributes to our understanding of these severe neuropathologies.

The proneural transcriptional factor Ascl1/Mash1 is an important regulator of neurogenesis in different regions of the embryonic nervous system^3^,^4^. In the developing cerebral cortex, Ascl1 regulates both early and late aspects of neurogenesis, including the division of radial glia progenitors^5^ and the radial migration of post-mitotic neurons^6^,^7^. Ascl1 achieves its diverse functions through direct transcriptional regulation of a large number of target genes. These include effector molecules controlling particular aspects of the neurogenic process, such as cell cycle progression or the reorganization of the cytoskeleton associated with neuronal migration^5^,^6^,^8^. Here we have identified the centrosome-associated protein Cenpj (centromere protein j, also known as CPAP) as a new and essential regulator of several crucial steps in the neurogenesis programme driven by Ascl1.

Mutations in the human *Cenpj* gene are responsible for primary autosomal microcephalies, including Seckel syndrome, characterized by severely reduced brain sizes^9^,^10^,^11^,^12^. Downregulation of Cenpj in HeLa cells causes centrosome duplication defects that lead to spindle malformation and modifies the orientation of the cleavage plane^13^,^14^. In mice, *Cenpj* deficiency produces a Seckel syndrome-like phenotype with a twofold smaller head^15^. Loss of *Cenpj* function in mouse fibroblasts results in centrosome defects causing mitotic spindle malformation and cell cycle arrest in G2/M, as well as genomic instability^15^.

In this study, we have examined the contribution of *Cenpj* to cortical neurogenesis in the mouse. We found that *Cenpj* has two distinct roles in progenitors and in post-mitotic neurons. The loss of *Cenpj* function in cortical progenitors leads primarily to a defect in centrosome formation that results in abnormal spindle orientation during mitosis. In neurons, the loss of *Cenpj* function compromises radial migration and morphology. Moreover, we found that *Cenpj* expression in the embryonic cortex is induced by Ascl1, and that *Cenpj* is the main regulator of centrosome biogenesis and microtubule stability downstream of *Ascl1* in the embryonic cerebral cortex.

## Results

### *Cenpj* is a transcriptional target of *Ascl1*

**Figure 1:**
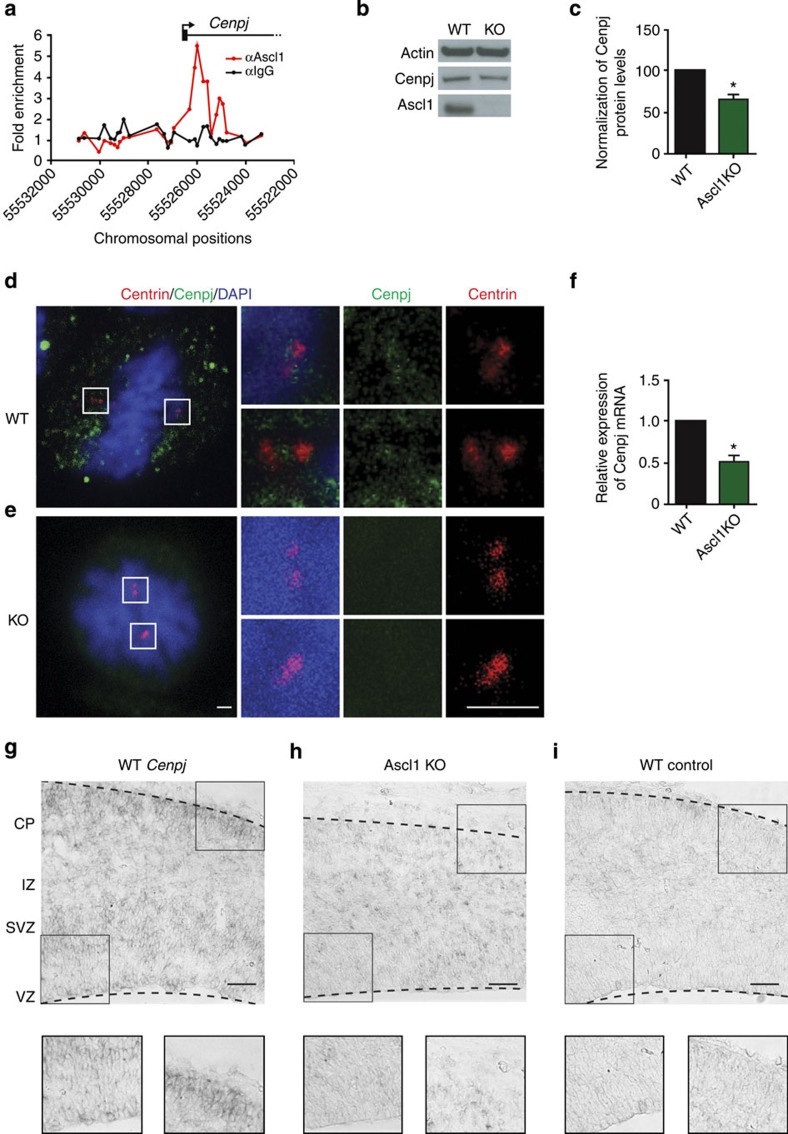
Ascl1 directly regulates *Cenpj*expression in the embryonic telencephalon. (**a**) Illustration of the Ascl1-binding signal in E14.5 telencephalic cells obtained by chromatin immunoprecipitation with promoter microarrays (chromatin immunoprecipitation (ChIP)-chip). The plot displays the ChIP enrichment ratio for Ascl1 (red) and control (black) samples in promoter regions. Black arrow indicates the transcription start site and direction of transcription. (**b**,**c**) Western blot analysis of Cenpj protein levels extracted from E14.5 *Ascl1* mutants compared with wild-type (WT) telencephalon. Data presented as mean±s.e.m., *n*=3, Student’s *t*-test, *P<0.05. (**d**,**e**) Immunohistochemistry for Cenpj on coronal sections of WT and *Ascl1* mutant E14.5 embryos. The Cenpj protein (**d**) is localized with the centrosome marker centrin in apical cortical progenitors and the signal is reduced in *Ascl1* mutant progenitors (**e**). Scale bars, 1 μm. (**f**) Quantitative PCR analysis of *Cenpj* transcripts extracted from E14.5 *Ascl1* mutants compared with WT telencephalon. Data presented as mean±s.e.m., *n*=3, Student’s *t*-test, *P<0.05. (**g**–**i**) *In situ* hybridization for *Cenpj* on coronal sections of the developing telencephalon at E14.5 from WT (**g**,**i**) and *Ascl1* knockout mouse (**h**) using an antisense *Cenpj* probe (**g**,**h**) and a control *Cenpj* sense probe (**i**). Expression of *Cenpj* was reduced in the ventricular zone and cortical plate of the *Ascl1* mutant cortex. Scale bar, 100 μm. DAPI, 4',6-diamidino-2-phenylindole.

In a previous study of the genes regulated by the proneural protein Ascl1 in the telencephalon of embryonic day 14.5 (E14.5) mice, Ascl1 was found to be bound near the transcription start site of the *Cenpj* gene^8^. To determine whether this binding event (Fig. 1a) results in the regulation of *Cenpj* by Ascl1, we examined the expression of *Cenpj* in the telencephalon of E14.5 wild-type and *Ascl1* null mutant embryos. Western blot (Fig. 1b,c) and immunocystochemistry analysis (Fig. 1d,e) showed that Cenpj protein is present at a reduced level in *Ascl1* mutant than in wild-type cortex. Cenpj is expressed in proliferating cortical progenitors throughout interphase and mitosis and is downregulated in *Ascl1* mutant cells throughout the cell cycle (Supplementary Fig. 1a). Quantitative PCR analysis showed a 50±5.7% reduction in *Cenpj* transcript in *Ascl1* mutant telencephalon (Fig. 1f). Analysis by *in situ* hybridization showed that *Cenpj* transcripts are present in the ventricular zone (VZ), subventricular zone (SVZ) and the cortical plate (CP) of the cerebral cortex in E14.5 wild-type embryos and are reduced in *Ascl1* mutant embryos (Fig. 1g–i; Supplementary Fig. 1b). Together, these results suggest that *Ascl1* regulates the *Cenpj* gene in the embryonic cortex and that it acts directly through interaction with a proximal regulatory element.

### *Cenpj* is required for centrosome biogenesis

To determine the contribution of *Cenpj* to cortical development downstream of Ascl1, we used an acute loss-of-function approach by RNA interference. We selected a short-hairpin RNA (shRNA) that specifically knocked down *Cenpj* and reduced its expression to ∼50% (Supplementary Fig. 2a), and co-electroporated *in utero* the VZ cells of the cerebral cortex at E14.5 with this *Cenpj* shRNA and a plasmid expressing green fluorescent protein (GFP) to visualize electroporated cells. As progenitors in the telencephalon of *Cenpj* conditional null mutant mice (that is, with complete loss of function) have been shown to undergo apoptosis^16^, we first examined the presence of apoptotic cells among GFP^+^ *Cenpj* knockdown cells (that is, with partial loss of function). There was no significant difference in numbers of activated caspase 3-positive cells between *Cenpj* shRNA- and control shRNA-electroporated brains, 1, 2 and 3 days after electroporation (Supplementary Fig. 2b,c).

**Figure 2:**
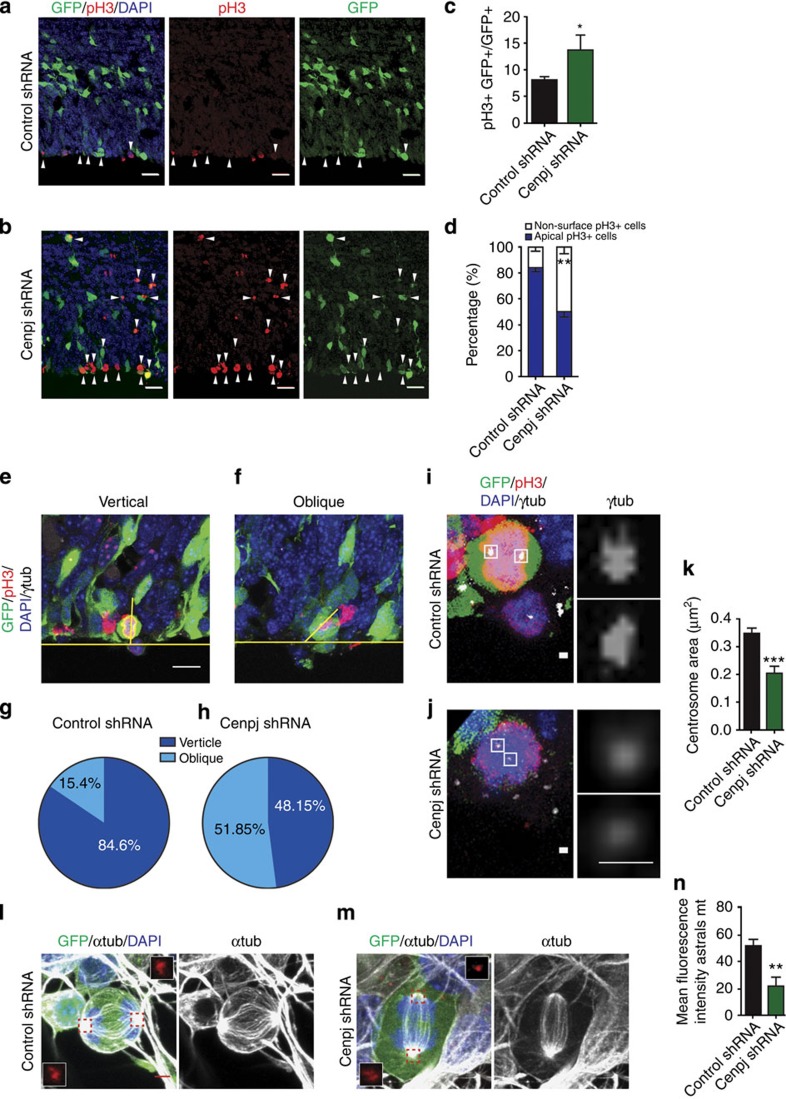
*Cenpj* is required for the vertical divisions of cortical progenitors. (**a**,**b**) Immunostaining with a pH3 antibody to identify cells in the M-phase of the cell cycle of embryonic cortices 1 day after *in utero* co-electroporation at E14.5 of GFP and a control scrambled shRNA (**a**) or a *Cenpj*-specific shRNA (**b**). Arrowheads point to cells with co-localized pH3 and GFP signals. Scale bar, 20 μm. (**c**) Quantification of GFP^+^ cells that also express pH3. Data presented here as the mean±s.e.m. from at least six sections prepared from three embryos obtained from two or three litters and >200 electroporated cells per embryo counted for each condition in this analysis and subsequent quantifications, unless stated otherwise. Student’s *t*-test *P<0.05. (**d**) Quantification of pH3^+^GFP^+^ cells located apically or scattered in the non-surface region 1 day after electroporation of the control or *Cenpj* shRNAs. Student’s *t*-test **P<0.01. (**e**,**f**) Examples of electroporated (GFP^+^) mitotic apical progenitors in metaphase with vertical (**e**) or oblique (**f**) cleavage planes determined by co-labelling strong pH3 mitotic cells and γ-tubulin to mark the centrosomes and the apical surface. Scale bar, 10 μm. (**g**,**h**) Quantification of vertical (60–90° angles with a ventricular surface) and oblique (30–60°) cleavage planes of mitotic apical progenitors 1 day after electroporation at E14.5 with control and *Cenpj* shRNAs. Control shRNA, *n*=22 cells; *Cenpj* shRNA, *n*=20 cells. Cells were analysed from at least six embryos obtained from three litters. (**i**,**j**) Electroporated (GFP^+^) mitotic (pH3^+^) apical progenitors labelled for centrosome marker γ-tubulin. Insets show higher magnification of the centrosomes. The centrosomes of *Cenpj* shRNA-electroporated cells (**j**) have a reduced size compared with those of control cells (**i**). Scale bars, 1 μm. (**k**) Quantification of centrosome size in apical cortical mitotic cells 1 day after electroporation. Student’s *t*-test ***P<0.001. (**l**,**m**) Electroporated (GFP^+^) progenitors labelled for centrosome marker Cdk5rap2 (red) and microtubule marker α-tubulin (grey). Insets show higher magnification of the centrosomes. *Cenpj* shRNA-electroporated cells generate asymmetric spindle and less astral microtubules. Scale bar, 3 μm. (**n**) Quantification of astral microtubules’ fluorescence intensity 2 days after electroporation. Student’s *t*-test **P<0.01. DAPI, 4',6-diamidino-2-phenylindole.

Cenpj is expressed in mitotic cells in the developing brain, suggesting that it may be required for normal proliferation of cortical progenitors. We therefore examined the divisions of electroporated cortical progenitors with an antibody against phosphohistone H3 (pH3) to identify cells in the M-phase of the cell cycle. We observed that the fraction of eletroporated cells in mitosis was increased among *Cenpj*-silenced cells compared with control electroporated cells (Fig. 2a–c). We also analysed the cell cycle profile of *Cenpj*-depleted cells 2 days after electroporation by Hoechst staining and flow cytometry (Supplementary Fig. 2d). The results confirmed that *Cenpj*-depleted cells accumulate in the M-phase (20.65±0.32% versus 16.73±1.1%, mean±s.e.m. throughout the text, *n*>200 cells). Moreover, *Cenpj*-silenced progenitors had an aberrant distribution with an increased number of pH3^+^ cells that were scattered in the VZ (Fig. 2b,d), and many of these cells expressed the basal progenitor marker Tbr2 (Supplementary Fig. 2e–g). The presence of scattered pH3^+^ cells in the VZ can be the result of a randomization of the cleavage plane of apical progenitors^17^,^18^,^19^,^20^. We therefore examined the cleavage plane of dividing apical progenitors when *Cenpj* was silenced, by double-labelling cells for pH3 and the centrosome marker γ-tubulin (Fig. 2e,f; Supplementary Fig. 2h). There was a strong increase in the fraction of *Cenpj*-silenced cells that divide with oblique cleavage planes and a reduction in cells dividing with a vertical plane, which were by far the most abundant in control electroporated cells (Fig. 2g,h). Randomization of the position of the mitotic spindle in *Cenpj*-silenced HeLa cells can result from a centrosome-formation defect^13^. We therefore examined centrosome formation in cortical VZ mitotic progenitors by measuring the centrosome area of γ-tubulin- or cdk5rap2-labelled centrosomes and observed a marked reduction in centrosome size in *Cenpj*-silenced cells (43±3.5%, *n*>30; Fig. 2i–k; Supplementary Fig. 2i,j), suggesting that *Cenpj* is required in apical progenitors for centrosome formation. Since *Cenpj* has also been shown to be required for centriole duplication in U2OS cells^13^,^14^, we counted centrioles in centrin–GFP co-electroporated cells. The fraction of cells with a single centriole was greatly increased when *Cenpj* was silenced (Supplementary Fig. 2k,l). Labelling with alpha tubulin to detect microtubules revealed that *Cenpj*-depleted cortical progenitors maintained a normal bipolar organization of the mitotic spindle, but that the quantity of astral microtubules was decreased in comparison with control progenitors. In addition, *Cenpj*-depleted cells presented asymmetric spindles with lower alpha tubulin fluorescence associated with a smaller centrosome at one pole (Fig. 2l–n).

The most parsimonious interpretation of the multiple defects observed in the *Cenpj*-silenced progenitor is that Cenpj is primarily required in cortical progenitors for centrosome duplication, and when this process is disrupted, progenitors are delayed in their cell cycle progression and accumulate in the M-phase, the quantity of astral microtubules is reduced, the orientation of the cleavage plane is randomized and daughter cells localize away from the ventricular surface. To determine whether the proliferation defects of *Cenpj*-depleted progenitors affect their rate of differentiation, we labelled electroporated cells for the early neuronal marker Tuj1, and found that *Cenpj* silencing resulted in fewer Tuj1+ neurons being generated 2 days after electroporation (Supplementary Fig. 2m–o).

### *Cenpj* is required for cortical neuron migration

**Figure 3:**
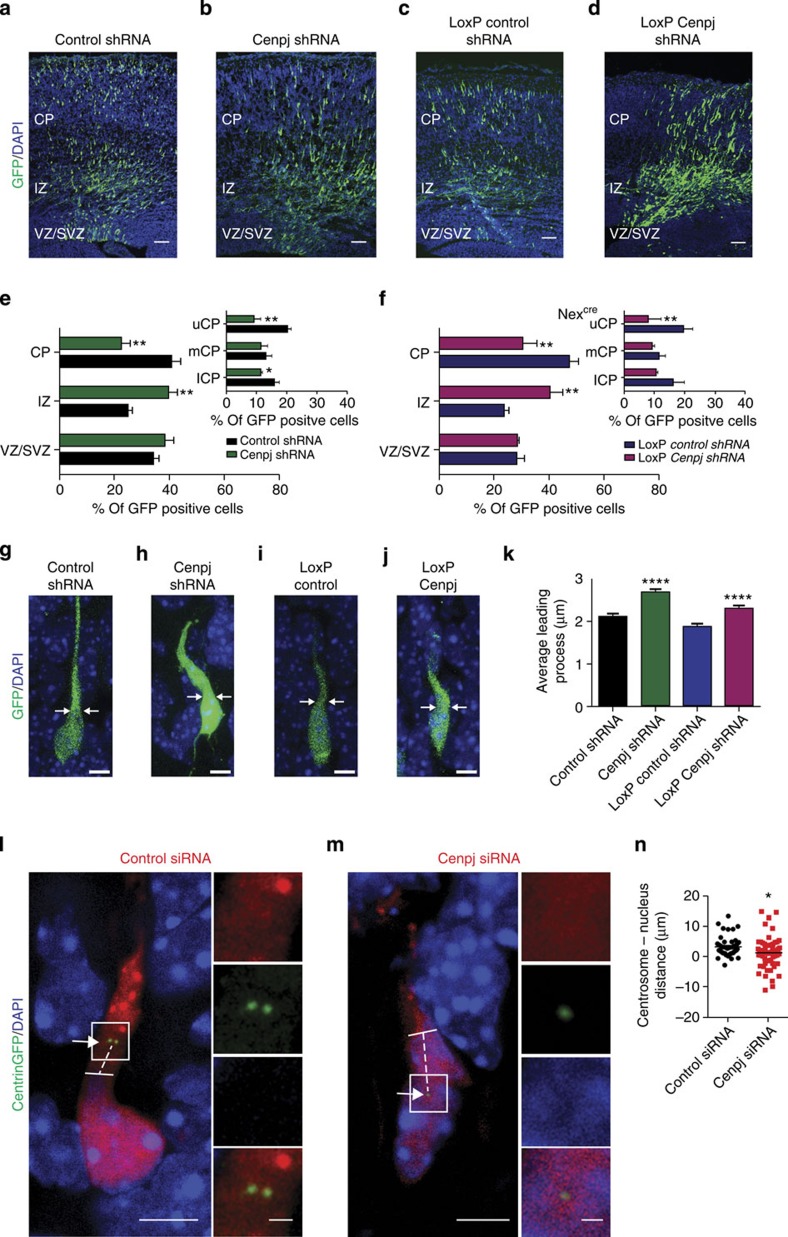
Silencing *Cenpj* alters neuronal migration and morphology. (**a**–**d**) Analysis of radial migration by GFP labelling of cortices 3 days after *in utero* co-electroporation at E14.5 with GFP and control shRNA (**a**), *Cenpj* shRNA (**b**). pCALSL control shRNA (**c**) and conditionally activable pCALSL_*Cenpj* shRNA (**d**). pCALSL control and_*Cenpj* shRNA were electroporated in Nex-cre embryos. Scale bar, 50 μm. (**e**,**f**) Quantification of the migration defect of neurons silenced for *Cenpj* in VZ progenitors (**e**) or in Nex+ neurons (**f**) by measuring the percentage of GFP^+^ cells that have reached the different zones of the cortex 3 days after electroporation. Cells silenced for *Cenpj* at both progenitor and neuronal stages accumulate more in the IZ and less in the upper CP than control cells. Student’s *t*-test *P<0.05, **P<0.01. (**g**–**j**) *Cenpj*-depleted neurons silenced at both progenitor (**h**) and neuronal stages (**j**) display abnormal enlargement of the leading process 3 days after electroporation compared with control neurons (**g**,**i**). Scale bar, 5 μm. (**k**) Quantification of the thickness of the leading process performed 3 days after electroporation, as described in the Methods section. *Cenpj*-depleted neurons have a thicker leading process. Three embryos analysed for each condition; control shRNA, *n*=177 cells; *Cenpj* shRNA, *n*=200 cells. Student’s *t*-test ****P<0.0001. (**l**,**m**) Analysis of the distance between the centrosome and the nucleus in migrating cortical neurons 3 days after *in utero* co-electroporation at E14.5 of control or *Cenpj* shRNAs together with pClG2-Centrin2-Venus for labelling of the centrioles and pCMV-RFP construct to mark electroporated cells. The nuclei are labelled with 4',6-diamidino-2-phenylindole (DAPI). Insets show higher magnification of the boxed areas with separate colour channels. Arrows point to the centrosome, dashed lines mark the distance between the centrosome and the tip of the nucleus, marked by a full line. Scale bar, 3 μm (main panels) and 1 μm (insets). (**n**) Quantification of the distance between the centrosome and the nucleus in migrating cortical neurons. Negative values correspond to centrosomes located below the tip of the nucleus. *Cenpj*-depleted neurons present more negative centrosome–nucleus distances than control neurons. Three embryos analysed for each condition; control shRNA, *n*=47 cells; *Cenpj* shRNA, *n*=60. Student’s *t*-test *P<0.05.

In addition to its expression and function in VZ progenitors, *Cenpj* is also expressed in post-mitotic neurons of the CP of the developing cerebral cortex (Fig. 1g; Supplementary Fig. 1b). Since a role of *Cenpj* in cortical neurons has not yet been studied, we investigated the consequence of *Cenpj* silencing in these cells by analysing GFP^+^-silenced cells 3 days after electroporation (Fig. 3a,b). *Cenpj* silencing resulted in a significant increase in the fraction of electroporated cells remaining in the intermediate zone (IZ) (39.3±3.4% *Cenpj* shRNA-electroporated versus 25.09±1.3% control shRNA-electroporated cells, *n*>200 cells), and a significant decrease in cells reaching the CP (22.55±3.1% versus 40.7±3.4%, *n*>200 cells; Fig. 3a,b,e). Double labelling for GFP and the neuronal marker Tuj1 two days after electroporation showed that 64% of electroporated cells in the SVZ and 85% in the IZ were neurons (Supplementary Fig. 3d,e). Moreover, *Cenpj*-silenced neurons that had reached the CP displayed aberrant morphologies (Fig. 3g,h; Supplementary Fig. 3g–i). While control electroporated neurons in the CP displayed a characteristic bipolar morphology with a straight and thin leading process, *Cenpj*-silenced neurons presented an enlarged leading process and thin processes protruding from the cell body.

To determine whether the abnormal migration of *Cenpj*-silenced cells results indirectly from the defects observed in VZ progenitors, or reflects a distinct, cell autonomous function of *Cenpj* in neurons, we silenced *Cenpj* specifically in post-mitotic neurons. For this, we used a construct that expresses the *Cenpj* shRNA in a Cre-dependent manner (floxed stop-*Cenpj* shRNA) in the cortex of *Nex-Cre* mice, which express Cre only in post-mitotic cortical neurons^21^. Cells that had been silenced for *Cenpj* at the post-mitotic stage displayed migration defects (40.27±2.8% *Cenpj* shRNA-electroporated versus 23.80±1.05% control shRNA-electroporated cells in the IZ, *n*>200 cells; Fig. 3c,d,f) and morphological defects identical to those of cells silenced at the progenitor stage (Fig. 3i–k), demonstrating that *Cenpj* has a function in post-mitotic neurons and is directly required for neuronal migration in the cortex, in addition to its role in centrosome duplication in progenitors. To determine whether the neuronal migration defect of *Cenpj*-silenced neurons is transient or persists at later times, we examined at postnatal 2 the position neurons electroporated at E14.5. A significant fraction of *Cenpj*-depleted cells remained mislocated (17.95±7.17% *Cenpj* shRNA- versus 48.70±3.1% control shRNA-electroporated cells in the upper CP (uCP), *n*>200 cells; Supplementary Fig. 3a–c), suggesting that *Cenpj* is required for the correct positioning of a large fraction of neurons in the CP.

### *Cenpj* promotes migration through microtubule destabilization

Migration of neurons in the CP is a cyclic process that includes a step of somal translocation during which the centrosome moves forward into the leading process and then pulls the nucleus to which it is connected by microtubules^22^. The increased thickness of the leading process of *Cenpj*-silenced neurons suggested that somal translocation might be impaired in these cells. To address this possibility, we co-electroporated a centrin–GFP construct together with *Cenpj* or control short interfering RNAs (siRNAs) and measured the distance between the centrosome and the tip of the nucleus (Fig. 3l,m) as an indication of centrosome–nucleus coupling. As expected, the centrosome was always found ahead of the nucleus in control experiments (Fig. 3l). In *Cenpj*-silenced neurons, however, not only the size but also the position of the centrosome were abnormal with some centrosomes located behind the front of the nucleus, suggesting that *Cenpj* is required for efficient coupling of the nucleus and centrosome in migrating neurons (Fig. 3m,n).

**Figure 4:**
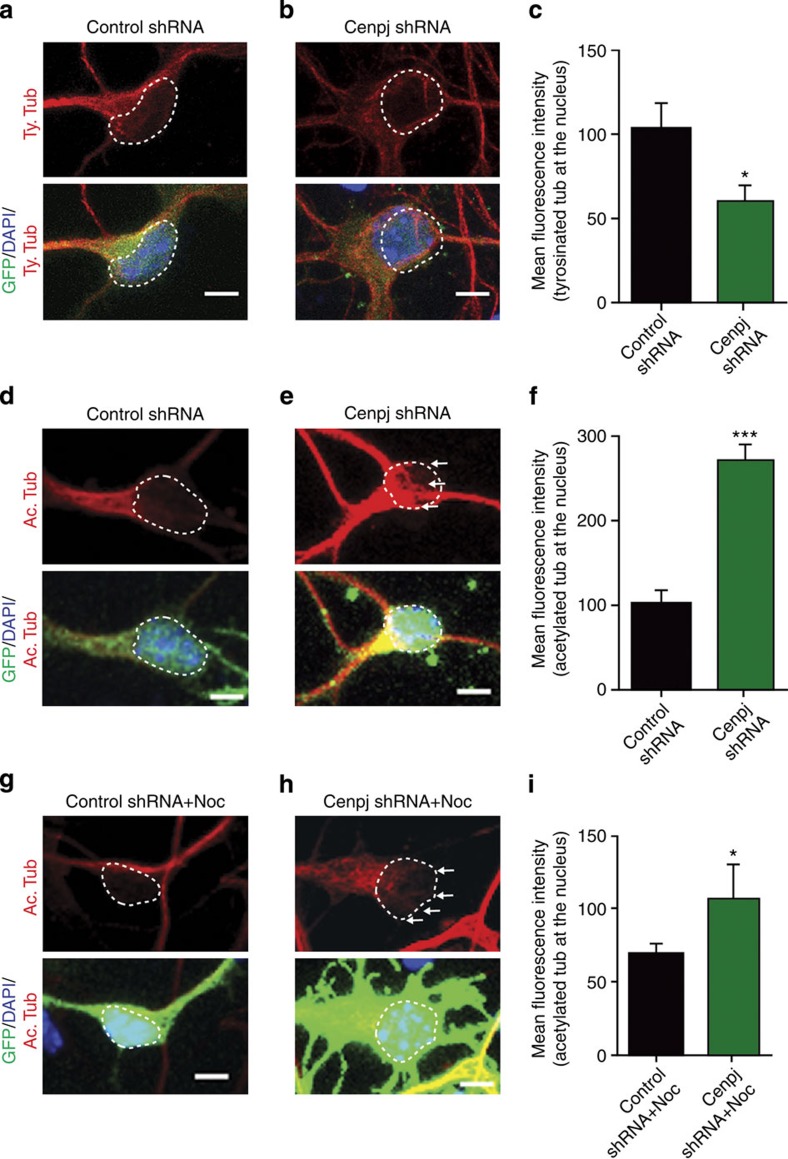
*Cenpj* silencing disrupts microtubule dynamics. (**a**,**b**,**d**,**e**) Analysis of dynamic tyrosinated microtubules (**a**,**b**) and stable acetylated microtubules (**d**,**e**) in neurons cultured *in vitro* for 3 days after *ex vivo* electroporation of GFP and control (**a**,**d**) or *Cenpj* shRNAs (**b**,**e**). White line shows 4',6-diamidino-2-phenylindole (DAPI) limits. Scale bar, 3 μm. (**c**,**f**) Quantification of the mean fluorescence intensity of tyrosinated tubulin (**c**) and acetylated tubulin (**f**) overlapping the nucleus labelled with DAPI. *Cenpj* silencing results in a reduction of dynamic microtubules and an increase in stable microtubules in the microtubule cage enveloping the nucleus. The analysis was performed in three independent cultures; control shRNA, *n*=44 cells; *Cenpj* shRNA, *n*=70 (**e**), control shRNA, *n*=60 cells; *Cenpj* shRNA, *n*=55 (**f**). Student’s *t*-test *P<0.05; ***P<0.001. (**g**,**h**) Effect of nocodazole on stable acetylated microtubules in neurons cultured *in vitro* for 3 days after *ex vivo* electroporation of GFP and control or *Cenpj* shRNAs (**g**,**h**). White line shows DAPI limits. Scale bar, 3 μm. (**i**) Quantification of acetylated tubulin labelling co-localized with the nucleus in nocodazole-treated cultures*. Cenpj* silencing results in an increase of stable microtubules in the microtubule cage compared with shRNA control-treated cells (arrows). Analysis of three independent cultures; control shRNA, *n*=40 cells; *Cenpj* shRNA, *n*=42. Student’s *t*-test *P<0.05.

The translocation of the nucleus requires optimal microtubule dynamics. Both excessive stability and instability of the microtubules can alter the distance between the nucleus and centrosome and the thickness of the leading process, and both can interfere with neuronal migration^23^,^24^,^25^,^26^,^27^. The abnormal position of the centrosome and altered morphology of the leading process in *Cenpj*-silenced neurons could therefore result from a defect in microtubule stability. In U2OS and Hela cells, *Cenpj* overexpression has been reported to destabilize microtubules^28^,^29^. To investigate whether *Cenpj* knockdown also affects microtubule stability, we analysed the distribution of stable and unstable microtubules in *Cenpj*-silenced neurons isolated from the E14.5 cortex and maintained for 3 days in culture. The perinuclear tubulin cage could be labelled in control neurons with an antibody against tyrosinated tubulin, which marks highly dynamic microtubules^30^ (Fig. 4a). In *Cenpj*-silenced neurons, the tyrosinated tubulin labelling was weaker throughout the cells, including over the nucleus (Fig. 4b,c). In contrast, labelling with an antibody against acetylated tubulin, which marks stable microtubules, was enhanced in *Cenpj*-silenced neurons compared with control neurons, and stable microtubule bundles were found abnormally located over the nucleus, suggesting that the perinuclear tubulin cage is more rigid in *Cenpj*-silenced neurons than in control neurons (Fig. 4d–f). To further examine microtubule stability in *Cenpj*-silenced neurons, we tested microtubule resistance to nocodazole-induced depolymerization^31^,^32^ in Tbr1-expressing cortical neurons electroporated *ex vivo* and maintained in culture for 3 days (Supplementary Fig. 4a,b). As expected, treatment of control cortical neurons with nocodazole reduced microtubule stability, as visualized by acetylated tubulin labelling (Fig. 4g). In contrast, *Cenpj*-silenced neurons displayed greater resistance to the nocodazole treatment, as shown by the accumulation of acetylated tubulin in the processes and the presence of aberrant microtubule protrusions over the nucleus (Fig. 4h,i). Conversely, *Cenpj* overexpression resulted in a destablization of microtubules, as indicated by a sharp reduction of acetylated tubulin staining (Supplementary Fig. 4,d).

To further investigate the role of Cenpj in microtubule dynamics, we co-electroporated shRNAs with an end-binding protein 1 (EB1)–GFP construct to analyse EB1 comet live production, as described previously^33^ (Supplementary Movies 1 and 2). *Cenpj* silencing significantly reduced the number of comets (Supplementary Fig. 4,g) and the speed of tracks (Supplementary Fig. 4f,h) in cultured neurons 2 days after electroporation. Together, these results suggest that *Cenpj* is required to promote microtubule destabilization, which is essential for proper nuclear–centrosome coupling and nucleokinesis in migrating cortical neurons.

### The PN2-3 protein domain is required for radial migration

**Figure 5:**
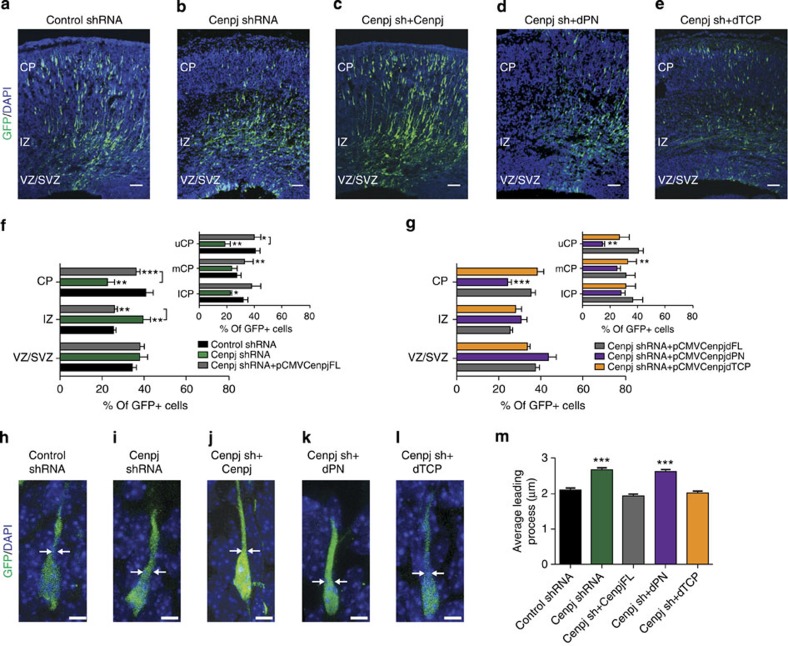
Microtubule-destabilizing domain of Cenpj is required for migration. (**a–e**) Analysis of radial migration in cortices 3 days after co-electroporation of GFP with a control shRNA (**a**), *Cenpj* shRNA alone (**b**), *Cenpj* shRNA together with a full-length *Cenpj* expression construct (pCMV-*Cenpj*, **c**), *Cenpj* shRNA with a truncated *Cenpj* construct lacking the microtubule-destabilizing domain PN2-3 (pCMV-*Cenpj* dPN2-3, **d**) or *Cenpj* shRNA with a truncated *Cenpj* construct lacking the domain TCP (pCMV-*Cenpj* dTCP, **e**). Scale bar, 50 μm. (**f**,**g**) Quantification of the migration defects of GFP^+^ cells in the different zones of the cortex. The migration defect of *Cenpj*-depleted cells is rescued by overexpression of full-length *Cenpj* (**f**) and *Cenpj* dTCP but not of *Cenpj* dPN2-3 (**g**). Student’s *t*-test *P<0.05; **P<0.01; ***P<0.001. (**h–l**) Analysis of leading process thickness in neurons in the CP co-electroporated with GFP and *Control shRNA* (**h**), with *Cenpj* shRNA (**i**) and *Cenpj shRNA* and *Cenpj* full length (**j**) or *Cenpj shRNA* and *Cenpj dPN* (K) or *Cenpj shRNA* and *Cenpj dTCP* (**l**). Scale bar, 5 μm. (**m**) Quantification of leading process thickness in GFP+ neurons. The leading process enlargement observed in *Cenpj*-depleted neurons is rescued by co-expression of *Cenpj* shRNA with *Cenpj* full length and *Cenpj dTCP* but not by *Cenpj dPN.* Three embryos analysed for each condition; control shRNA, *n*=177 cells; *Cenpj* shRNA, *n*=200 cells; *Cenpjsh+Cenpj* FL, *n*=157 cells; *Cenpj* shRNA *+Cenpj dPN2-3*, *n*=274 cells; *Cenpj* shRNA+*Cenpj* dTCP, *n*=211 cells. Student’s *t*-test ***P<0.001. DAPI, 4',6-diamidino-2-phenylindole.

The conserved PN2-3 domain of Cenpj has previously been shown to be both necessary and sufficient for the microtubule destabilization activity of the protein^34^. This domain has also been shown to be involved in centrosome formation^13^. To determine whether the PN2-3 domain contributes to *Cenpj* function in neuronal migration, we tested the capacity of a mutant version of Cenpj with a deleted PN2-3 domain (*dPN2-3*) to rescue the *Cenpj* knockdown phenotype when co-expressed with the *Cenpj* shRNA (Fig. 5d). As expected, co-electroporation of *Cenpj* shRNA with a full-length shRNA-resistant version of *Cenpj* fully rescued both the migration and morphological defects of *Cenpj*-silenced neurons (*FL*; Fig. 5a–c,f,h–j). In contrast, expression of shRNA-resistant *Cenpj dPN2-3* failed to rescue the defective migration (23.25±1.2% versus 40.7±3.4% in the CP in control experiments, *n*>200 cells) and the abnormal leading process of *Cenpj*-silenced neurons, which were both as severe as for neurons electroporated with *Cenpj* shRNA alone (Fig. 5d,g,l,m). The PN2-3 domain is therefore essential for *Cenpj* function in cortical neuron migration. Another domain of the Cenpj protein, T complex protein 10 (TCP), has been shown to be required for the centrosome duplication function of the protein^13^, but to be dispensable for its microtubule destabilization function^13^,^29^. To determine whether the TCP domain contributes to the activity of Cenpj in radial migration, we co-electroporated the *Cenpj* shRNA with a version of *Cenpj* that carries a deletion of this domain (*dTCP*). *Cenpj dTCP* rescued the migratory behaviour and morphological defect of silenced neurons as efficiently as full-length *Cenpj* as shown in Fig. 5g (38.78±2.9% versus 40.7±3.4% in the CP in control experiments, *n*>200 cells) and Fig. 5m. We also examined whether the different Cenpj protein domains rescued the defects in microtubule stability and centrosome position observed in *Cenpj*-deficient neurons (Supplementary Fig. 5). Interestingly, only dTCP could rescue microtubule stability effects (Supplementary Fig. 5a–c) and centrosome–nucleus coupling (Supplementary Fig. 5d–f). However, neither TCP nor PN2-3 rescued the centrosome size or centriole number defects of *Cenpj*-deficient progenitors (Supplementary Fig. 2i–2l). Together, these results suggest that *Cenpj* promotes nuclear translocation and neuronal migration in the developing cortex by destabilizing microtubules through the activity of its PN2-3 domain, independently of its role in centrosome formation.

### *Cenpj* is an effector of Ascl1

**Figure 6:**
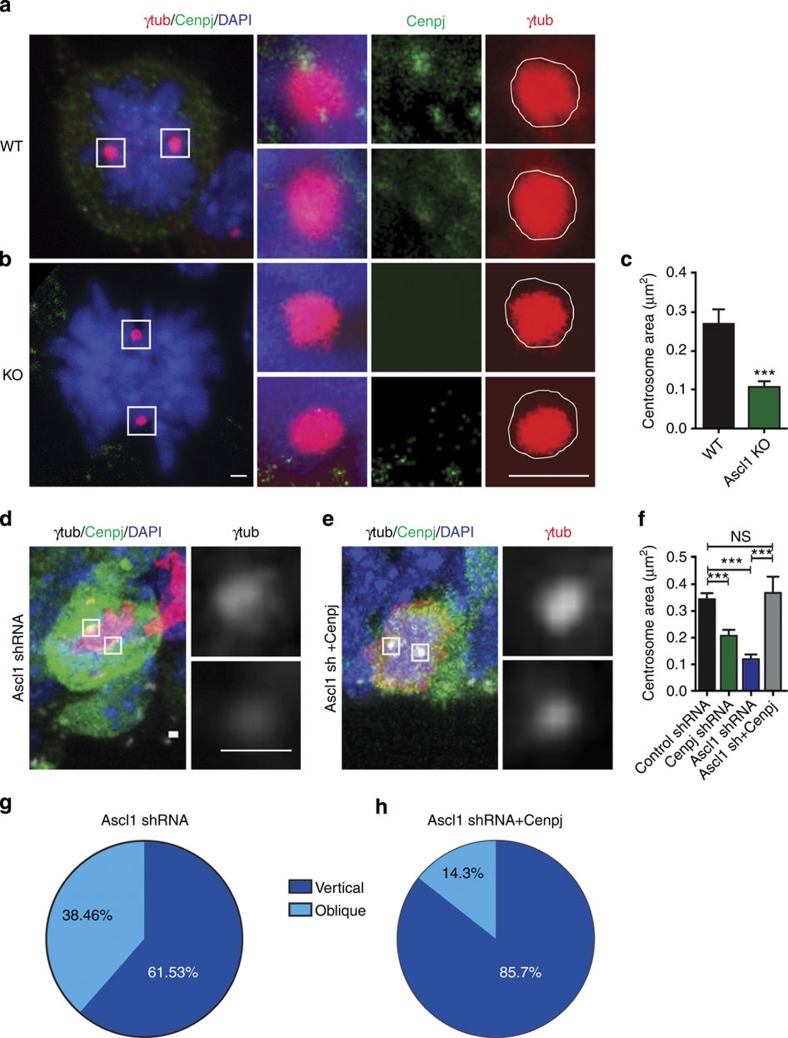
*Cenpj* is the main effector of *Ascl1*for centrosome biogenesis. (**a**,**b**) Labelling of centrosomes in apical cortical progenitors from wild-type (WT) (**a**) or *Ascl1* null embryos (**b**) with an antibody to γ-tubulin. Insets to the right show higher magnification of the boxed areas with separate colour channels. Scale bars, 1 μm. (**c**) Quantification of the area of the centrosomes in WT and *Ascl1* null embryos labelled with an antibody to γ-tubulin. Three embryos analysed for each condition. Student’s *t*-test ***P<0.001. (**d**,**e**) Analysis of the centrosome area of GFP+-dividing apical progenitors electroporated 1 day earlier *in utero* with *Ascl1* shRNA (**d**) or an *Ascl1* shRNA plus a pCMV-*Cenpj* construct (**e**) labelled for GFP (green), pH3 (red) and γ-tubulin (grey). Insets to the right show higher magnification of the boxed areas with γ-tubulin (grey) channels. Scale bars, 1 μm. (**f**) Quantification of the area of the centrosomes in control shRNA-electroporated progenitors, *Cenpj shRNA*, *Ascl1* shRNA and *Ascl1* shRNA+pCMV-Cenpj-electroporated progenitors, labelled with γ-tubulin. The centrosomes are smaller in *Ascl1*-silenced progenitors and this defect is rescued in *Ascl1*-silenced cells co-electroporated with a Cenpj expression vector. Student’s *t*-test ***P<0.001. (**g**,**h**) Percentage of cells electroporated with *Ascl1* shRNA (**g**) and *Ascl1* shRNA+pCMV-*Cenpj* (**h**) having a vertical (60–90°) or oblique (30–60°) cleavage angle. *Ascl1* shRNA, *n*=33 cells; *Ascl1* shRNA*+Cenpj*, *n*=24 cells. Cells were analysed from at least six embryos obtained from three litters. DAPI, 4',6-diamidino-2-phenylindole; NS, not significant.

The foregoing data demonstrate that *Cenpj* has important roles in both progenitor cells and neurons during cortical development. Since *Cenpj* expression in the embryonic cortex is regulated by the proneural protein Ascl1 (Fig. 1), *Cenpj* might contribute significantly to *Ascl1* functions in cortical development. To address this possibility, we compared the defects caused by *Ascl1* loss of function in cortical progenitors with those displayed by *Cenpj*-silenced progenitors. Labelling for γ-tubulin and measuring the centrosome area showed that centrosomes in mitotic cortical cells were smaller in *Ascl1* mutant progenitors than in wild-type progenitors (60.36±6%, *P*<0.001, Student’s *t*-test, *n*>30 per condition; Fig. 6a–c). To determine whether *Cenpj* is the main effector of *Ascl1* in the regulation of centrosome formation, we overexpressed *Cenpj* in *Ascl1*-silenced apical progenitors and we found that this fully corrected the size of the centrosome (Fig. 6d–f).

The cleavage plane of cortical apical progenitors has previously been shown to be essentially random in *Ascl1* mutant E14.5 embryos instead of mostly vertical in wild-type embryos, similar to the defects observed in *Cenpj*-silenced progenitors (Figs 2f and 6g)^5^. To determine whether *Cenpj* contributes to the function of *Ascl1* in regulating spindle orientation, we overexpressed *Cenpj* in *Ascl1*-silenced apical progenitors. We found that this fully corrected the abnormal orientation of the progenitor divisions to mostly vertical angles (Fig. 6h).

**Figure 7:**
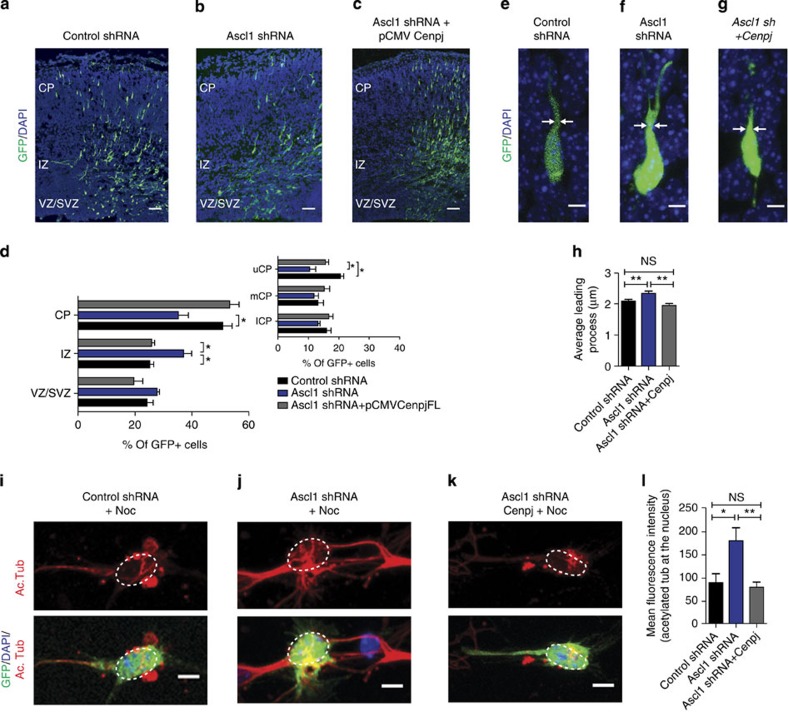
*Cenpj* is the main effector of *Ascl1*for microtubule regulation. (**a–c**) Analysis of radial migration 3 days after electroporation of E14.5 cortices with GFP and a control shRNA, *Ascl1* shRNA or *Ascl1* shRNA+pCMV-*Cenpj*. Scale bar, 50 μm. (**d**) Quantification of the migration of GFP^+^ cells in the different zones of the cortex. The accumulation of *Ascl1*-silenced cells in the IZ but not their depletion from the uCP was rescued by overexpression of *Cenpj.* Student’s *t*-test *P<0.05 (**e–g**). Abnormal leading-process enlargement of *Ascl1*-silenced neurons in the CP. Scale bar, 5 μm. (**h**) Quantification of leading process thickness. Three embryos analysed for each condition; control shRNA, *n*=177 cells; *Ascl1* shRNA, *n*=128 cells; *Ascl1* shRNA+pCMV-*Cenpj*, *n*=239 cells. Student’s *t*-test **P<0.01. (**i–k**) Nocodazole treatment of cultured neurons 3 days after electroporation. *Ascl1* silencing preserved a higher level of acetylated tubulin labelling in the cell body and leading process than control shRNA, and overexpression of *Cenpj* restored the acetylated tubulin-labelling intensity. Scale bar, 5 μm. (**l**) Quantification of acetylated tubulin labelling co-localized with the nucleus. Three independent cultures analysed for each condition; control shRNA, *n*=40 cells; *Ascl1* shRNA, *n*=53; *Ascl1* shRNA+pCMV-*Cenpj*, *n*=37. Student’s *t*-test *P<0.05; **P<0.01. DAPI, 4',6-diamidino-2-phenylindole; NS, not significant.

*Ascl1* silencing in cortical progenitors results 3 days later in accumulation of their progeny in the IZ and in their depletion from the uCP^6^ (Fig. 7a,b,d), a defect similar to that observed with *Cenpj*-silenced neurons. Also similar to *Cenpj*-silenced cells, *Ascl1*-silenced neurons in the CP exhibit an enlargement of the leading process (Fig. 7e,f) and microtubules also appear bundled, as shown by the accumulation of acetylated tubulin over the nucleus after nocodazole treatment (Fig. 7i,j). Co-electroporation of *Cenpj* with the *Ascl1* shRNA corrected the abnormal accumulation of *Ascl1*-silenced cells in the IZ (37.0±2.8% *Ascl1* shRNA versus 25.7±1.13% *Ascl1* shRNA+pCMV-*Cenpj*-electroporated versus 25.09±1.37% control shRNA-electroporated cells, *n*>200 cells; Fig. 7c,d). The abnormal morphology of the leading process of *Ascl1*-silenced cells (Fig. 7g,h) and the accumulation of acetylated tubulin staining over the nucleus were also corrected by overexpression of *Cenpj* (Fig. 7k,l). However, the migration of *Ascl1*-silenced neurons to the upper part of the CP was not corrected by overexpression of *Cenpj* (Fig. 7d), suggesting that another gene regulated by Ascl1 is required for this later phase of migration.

Altogether, these results demonstrate that *Cenpj* regulates several crucial steps of neurogenesis in the cerebral cortex downstream of *Ascl1*, including centrosome biogenesis, the orientation of progenitor cleavage planes and the destabilization of microtubules and nuclear translocation during neuronal migration.

## Discussion

The transcription factor Ascl1 is expressed in progenitors of the embryonic cerebral cortex^4^ and has specific functions in cortical neurogenesis, although it is not directly involved in the neuronal commitment and differentiation of progenitors in this brain region, which is a role taken by another proneural protein, Neurog2 (refs 35, 36). Ascl1 is required for the proper orientation of the progenitor divisions in the VZ^5^ and for the migration of cortical neurons through the CP^6^. The pathways that mediate these functions of Ascl1 have begun to be characterized. Here we identify the microcephaly protein *Cenpj* as a target of Ascl1 involved in multiple steps of cortical neurogenesis. Below, we discuss first the function of *Cenpj* in dividing cortical progenitors and then its role in migrating cortical neurons.

Our demonstration that *Cenpj* is required in cortical progenitors for centrosomes to reach a normal size is in agreement with previous studies examining the effect of *Cenpj* silencing in HeLa, U2OS and mouse embryonic fibroblast cells (MEFs). These earlier studies also showed that the centrosome biogenesis defect leads to mitotic spindle abnormalities^13^,^14^,^16^, which we also observed in *Cenpj*-depleted cortical progenitors in this study. Similarly, the analysis of mice mutant for the centrosome-associated proteins Treacle and Nde1 and the cell cortex-associated protein Lgn have shown that randomization of the plane of progenitor divisions results in mitotic progenitors being located away from the apical cortical surface^17^,^18^,^20^. Abnormal angles of progenitor divisions are likely to be also at the origin of the displaced dividing *Cenpj*-silenced progenitors. A large fraction of these scattered progenitors acquire a basal progenitor fate, as shown by Tbr2 expression and as reported previously^37^. It seems less likely that *Cenpj* silencing results in an increased proliferation of basal progenitors because this would result in an increase in neuronal production, and we observe instead a decrease.

*Cenpj* hypomorphic mice present a reduction in brain size, but not an increase in apoptosis in the dorsal telencephalon^15^. Similarly, we did not observe an increase in cell death following partial loss of function of *Cenpj* in the developing cerebral cortex by *shRNA*-mediated knockdown. However, embryos with a complete elimination of *Cenpj* present a widespread, p53-dependent cell death phenotype^16^. We therefore suggest that the severity of the phenotype resulting from *Cenpj* deficiency is dependent on the level of Cenpj protein expressed.

Altogether, based on our data and on previous studies in different systems, we propose that the primary role of *Cenpj* in progenitors is in the elongation of the procentrioles during centrosome duplication, as well as in the recruitment of the pericentriolar material as a scaffolding protein^13^,^14^,^38^,^39^. As a consequence of *Cenpj* depletion, the centrioles are shorter and the pericentriolar material is not recruited properly, resulting in a smaller and fragmented centrosome. This primary centrosome biogenesis defect leads to abnormal centrosome duplication, spindle malformation and mitotic cleavage randomization.

While the role of *Cenpj* in regulating centrosome formation and mitotic spindle orientation had been previously reported in cultured cells, its involvement in cell migration that we report in cortical projection neurons is novel. The role of *Cenpj* in the radial migration of cortical neurons involves the destabilization of the microtubule cage that surrounds the nucleus, thus promoting efficient centrosome–nucleus coupling. This is clearly independent from the role of *Cenpj* regulation of centrosome size in progenitors, because the migratory defect is observed specifically when *Cenpj* is silenced in neurons. Moreover, it is rescued by a version of Cenpj that lacks the TCP domain, involved in centrosome formation, while it is not rescued by a version lacking the microtubule-destabilizing domain PN2-3 (ref. 13). The PN2-3 domain inhibits microtubule assembly *in vitro* by binding and sequestering single α/β-tubulin dimers^28^,^40^. A recent study also reported cortical neuron migration defects in *Cenpj* null mutant embryos, in which progenitor cell death had been rescued by p53 deletion, manifested by the formation of small cortical heterotopias^41^. Cenpj thus joins the list of microtubule-destabilizing proteins that have been shown to be required for maintenance of a dynamic microtubule cage, the regulation of the thickness of a leading process and proper positioning of the centrosome with respect to the nucleus, including the microtubule-severing proteins katanins and the microtubule disassembly proteins stathmins^26^,^42^,^43^,^44^. Other microcephaly proteins, such as ASPM and Ndel1, also have essential roles in progenitor divisions and post-mitotic neurons^45^,^46^,^47^,^48^, and have been shown to act by interacting with motor proteins. Ndle1 interacts with Lis1 and the dynein complex^49^, while the *Caenorhabditis elegans* ASPM orthologue ASPM-1 interacts with dynein^50^ and the *Drosophila* ASPM orthologue Asp interacts with myosin II^51^. Whether Cenpj also influences motor protein activity remains to be investigated.

Analysis of the Drosophila mutant for the *Cenpj* homologue *Sas-4* did not reveal any brain-organization defects^52^. We show here that a deficit in *Cenpj* results in abnormal positioning of a significant fraction of cortical neurons at postnatal stages. Whether loss of *Cenpj* also results in cortical connectivity defects and abnormal brain function is an important question that remains to be addressed.

The regulation of *Cenpj* expression in the cerebral cortex by *Ascl1* and the similarities of the migratory defects of *Cenpj*- and *Ascl1*-deficient neurons suggested that *Cenpj* might mediate some aspects of *Ascl1* function in neuronal migration. Indeed, co-expression of *Cenpj* with an *Ascl1* shRNA rescued the abnormal accumulation of *Ascl1*-silenced neurons in the IZ, thus establishing the importance of *Cenpj* activity in the pro-migratory programme driven by *Ascl1*. However, the rescue of *Ascl1*-silenced neurons was not complete since cells did not migrate to the uCP as efficiently as control cells. This result was expected since *Ascl1* also regulates the cortical expression of Rnd3, a constitutively active small guanosine triphosphate-binding protein that promotes cortical neuron locomotion in the CP by inhibiting RhoA activity and destabilizing the actin cytoskeleton^6^. Together, our studies show that Ascl1 controls neuronal migration by regulating both the actin and microtubule cytoskeletons, via Rnd3 and Cenpj, respectively.

The function of *Cenpj* at two crucial steps of cortical development likely explains the long-term consequences of *Cenpj* deficiency in the formation and function of the cerebral cortex. Our work provides insights into the mechanisms of *Cenpj* function and regulation. It implicates the proneural factor Ascl1 and the microcephaly protein Cenpj in neuronal migration, thus improving our understanding of human microcephalies and microlissencephalies.

## Methods

### Animals

Mice were housed, bred and treated according to the guidelines approved by the Home Office under the Animal (Scientific procedures) Act 1986. The following mouse lines were used and genotyped, as described previously: *Ascl1*^*delta*^^53^; *Nex*^*Cre*^^21^.

### Cell culture

Mouse embryonic teratocarcinoma P19 were cultured in high-glucose DMEM (Invitrogen) supplemented with 10% fetal bovine serum, 2 mM glutamine and 1% penicillin/streptomycin. NS5 mouse cells and cortical progenitors from E14.5 wild-type or *Ascl1* mutant were plated onto uncoated tissue culture plastic with the addition of 2 μg ml^−1^ laminin (Sigma) to the medium (Euromed) supplemented with epidermal growth factor and fibroblast growth factor (both at 10 ng ml^−1^; Peprotech) incubated at 37 °C under 5% CO_2_ atmosphere. P19 and NS5 cells were plated in 24-well plates and transfected with Lipofectamine 2000 reagent according to the manufacture’s protocol (Invitrogen). Twenty-four hours post transfection, cells were collected for protein or RNA extraction.

### *Ex vivo* cortical electroporation

*Ex vivo* electroporation was performed on injected mouse embryo heads. The electrical parameters were: 50 V, 50 ms length, 5 pulses and 1 s interval. After electroporation, brains were dissected in Gey’s buffer (Sigma) and cut coronally (300 μm) with a tissue chopper (McIlwain). Slices were transferred onto sterilized culture plate inserts (0.4-μm pore size; Millicell-CM, Millipore) and cultivated in complete Neurobasal containing Neurobasal medium (Invitrogen) supplemented with 1% B27, 1% N2, 1% glutamine, 1% penicillin/streptomycin and 1% fungizone. Slices were cultivated for 1 day followed by dissection of the GFP+ portion of the cortex under a GFP binocular. Cells were dissociated in HBSS (Sigma) mechanically with round Pasteur pipettes, seeded on poly-Dlysine- (10 μg ml^−1^, Sigma) and laminin (10 μg ml^−1^, Sigma)-coated wells (Lab-Tek, Chamber Slide, four-well Permanox slides) and cultured in complete Neurobasal medium for neuronal differentiation or cultured in proliferation medium (Euromed) supplemented with epidermal growth factor and fibroblast growth factor (both at 10 ng ml^−1^; Peprotech).

### *In situ* hybridization

To determine the expression profile of *Cenpj*, we performed messenger RNA (mRNA) hybridization directly on tissue sections. Embryonic brains were fixed overnight in 4% paraformaldehyde (PFA) and transferred into 20% sucrose. After overnight incubation, the brains were embedded in optimal cutting temperature compound (OCT) (VWR International) and coronally cryosectioned (12 μm). The sections were post-fixed with 4% PFA and treated with 0.25% acetic anhydride and triethanolamine to acetylate the amino groups on proteins and reduce the background. After a pre-hybridization step at 70 °C, the sections were incubated overnight at 70 °C with digoxigenin-labelled RNA probes complementary to target mRNA. To detect the riboprobes that bound to the tissue, sections were incubated overnight at 4 °C with an anti-digoxigenin antibody conjugated with alkaline phosphatase. The signal was then revealed by adding nitro blue tetrazolium 5-bromo 4-chloro 3-indolyl phosphate (Roche), the substrate of alkaline phosphatase, to the sections.

### *In utero* electroporation and tissue processing

*In utero* electroporation of the cerebral cortex was performed in anesthetized time-pregnant mice (14.5 days), as previously described^6^. Briefly, one telencephalic embryonic ventricle for each embryo was injected with 1 μl of 1 μg μl^−1^ endotoxin-free DNA. Cortices were electroporated with five 50-ms electrical pulses at 30 V with 1-s intervals using 5-mm platinum electrodes (Harvard Apparatus). At the desired time point after electroporation, mice were culled and embryos were processed for tissue analyses. Embryonic brains were fixed in 4% PFA overnight and then placed in 20% sucrose/PBS overnight. Embryonic brains were then embedded in OCT and frozen before sectioning using a cryostat. After immunostaining, all images were acquired with a laser scanning confocal microscope (Leica SP5). Cell counts were performed using Fiji (ImageJ). The different cortical zones namely VZ, SVZ, IZ and CP were identified based on cell density visualized with 4',6-diamidino-2-phenylindole (Invitrogen) nuclear counterstaining. The CP was equally subdivided in three bins called uCP, medium CP and lower CP. Tuj1 neuronal marker staining co-localized with GFP was used to further identify newborn neurons in the VZ and SVZ, and migrating neurons in the IZ and CP.

### Immunostaining

After washing in PBS, sections were treated with PBS—0.1%, Triton X-100—10% serum for 30 min. They were then incubated overnight at 4 °C with the following primary antibodies diluted in the blocking buffer: mouse anti-acetylated tubulin 1:100 (Sigma); mouse anti-α-tubulin (1:100, Sigma-Aldrich); rabbit anti-Cenpj: 1:200 (Proteintech); rabbit anti-Cdk5rap2 (1:100, Milipore); rabbit anti-centrin: 1:100 (Milipore); chicken anti-GFP (1:700, Millipore); rabbit anti-pH3 (1:200, Upstate); mouse anti-γ-tubulin (1:100, Abcam) and mouse anti-tyrosinated tubulin 1:100 (Sigma). Sections were then incubated with appropriate fluorescent secondary antibodies. To detect Cenpj, a step of antigen retrieval with citrate was performed before the blocking (sodium citrate pH=6 for 20 min at 90 °C).

### Nocodazole treatment

After two days *in vitro*, cultures were treated for 30 min with 6 μM nocodazole (Sigma) or dimethylsulphoxide (Sigma) and fixed with 4% formaldehyde at 37 °C for 2 min followed by 3 min MeOH at −20 °C, as described previously^31^.

### Quantification of immunohistochemical data

Immunofluorescence mean of acetylated or tyrosinated microtubules from the nucleus area was measured with ImageJ. Centrosome diameter was measured in each centrosome of the mitotic cell with ImageJ and the area was quantified (μm^2^). Leading process thickness was measured by drawing a transversal line at the portion of the leading process adjacent to the nucleus with ImageJ. Mitotic spindle orientation and morphology was measured in high-magnification confocal images of cortical sections using ImageJ orthogonal three-dimensional viewer of hyperstacks. Taking into consideration the ventricle lining labelled with *γ*-tubulin and using the three-dimensional viewer to ensure that the centrosome analysed belongs to the metaphase cell examined, we measured the angle between the cleavage plane and the ventricle. Immunofluorescence mean of the astral microtubule area was measured with ImageJ. Spindles were classified as bipolar or monopolar, symmetric or asymmetric, as described previously^13^.

### Plasmids constructs and siRNAs

*Cenpj* shRNA (5′- TCCGACTGGATGCCTGGAAGAGAGCAGAA -3′), Ascl1 shRNA (5′- AGAAGATGAGCAAGGTGGAGACGCTGCGC -3′) control shRNA (5′- GCACTACCAGAGCTAACTCAGATAGTACT -3′) plasmids were purchased from Origene. *Cenpj* siRNA (5′- GGAGUAAAAUUGACUUCGA -3′) and Silencer Negative Control No. 1 siRNA (catalogue number AM4636) were purchased from Ambion. To allow conditional expression of control shRNA and *Cenpj* shRNA using the Cre-LoxP system, sequences were cloned into pCALSL mir-30 vector (Addgene plasmid 13786), as previously described^54^, and verified by western blots (Supplementary Fig. 3f). EB1–GFP plasmid was purchased from Addgene (plasmid 17234).

### Real-time PCR

*Cenpj* mRNA expression was quantified by quantitative real-time PCR in control and shRNA-treated samples. RNAs were extracted from P19 cells or whole-telencephalon tissue using TRIzol reagent (Invitrogen), followed by a classical phenol/chloroform separation. The aqueous phase was mixed with isopropanol to precipitate the nucleic acid, which was then washed with 70% ethanol and resuspended in RNAse-free water. A clean-up step was then carried out using the RNeasy mini kit protocol (Qiagen). Purified RNAs were then reverse-transcribed into cDNA using the Applied Biosystems kit. Real-time PCR was performed using Taqman probes according to the manufacturer’s protocol (TaqMan gene expression assays, Applied Biosystems). The Taqman probes for *Cenpj* and for the endogenous control, β-actin, was purchased from Applied Biosystems. The 7500-system software provided by Applied Biosystem was used to run the PCR and analyse the data.

### Statistical analysis

Data are expressed as mean±s.e.m. Statistical analysis was performed using an unpaired two-tailed Student’s *t*-test with Prism software, version 6. *P<0.05, **P<0.01, ***P<0.001, ****P<0.0001.

### Western blotting

To determine the efficiency of the shRNAs, we analysed the expression levels of the protein of interest in control and shRNA-treated samples, by SDS–polyacrylamide gel electrophoresis protein separation and immunoblotting. Protein extracts from P19 cells or whole-telencephalon tissue were obtained by lysis with the Promega passive lysis buffer complemented with a cocktail of protease inhibitors (Roche). Western blotting was performed using standard protocols^55^. The primary antibodies were diluted in the blocking solution at the following concentrations: rabbit anti-Cenpj: 1:400 (Proteintech), rabbit anti-actin, 1:1,000 (Sigma, A2066) and rabbit anti-alpha tubulin, 1:1,000 (Sigma) and mouse anti-Mash1, 1:1,000, BD bioscience. Horseradish peroxidase-conjugated secondary antibodies were used as follows: goat anti-rabbit, 1:200 (Dako, P0449) and goat anti-mouse, 1:1,000 (Sigma, A8924). Uncropped scans of Fig. 1b and Supplementary Fig. 3f blots are shown in Supplementary Fig. 6.

## Author contributions

P.P.G. and F.G. conceived all the experiments, analysed the data and wrote the manuscript. Together with P.P.G., J.D.-A. and I.C.-E. participated in carrying out experiments requested by reviewers. D.C. contributed with ChIP-chip data. D.B. helped with live imaging experiments and analysis of EB1-comets. All authors discussed the results and commented on the manuscript.

## Additional information

**How to cite this article:** Garcez, P. P. *et al*. Cenpj/CPAP regulates progenitor divisions and neuronal migration in the cerebral cortex downstream of Ascl1. *Nat. Commun.* 6:6474 doi: 10.1038/ncomms7474 (2015).

## Supplementary Material

Supplementary Information Supplementary Figures 1-6, Supplementary Methods.

Supplementary Movie 1 EB1-GFP live comets in a co-electroporated Control shRNA neuron after two days in vitro, related to Supplementary Fig. 4e-h.

Supplementary Movie 2 EB1-GFP live comets in a Cenpj-depleted neuron after two days in vitro, related to Supplementary Fig. 4e-h
