# Supplementary material for: Cenpj/CPAP regulates progenitor divisions and neuronal migration in the cerebral cortex downstream of Ascl1
Source: Nat Commun. 2015 Mar 10;6:6474. doi: 10.1038/ncomms7474 (PMC4366522; doi:10.1038/ncomms7474)
Supplement: Supplementary Information — Supplementary Figures 1-6, Supplementary Methods. [file ncomms7474-s1.pdf]

SUPPLEMENTARY FIGURES

Supplementary Figure 1

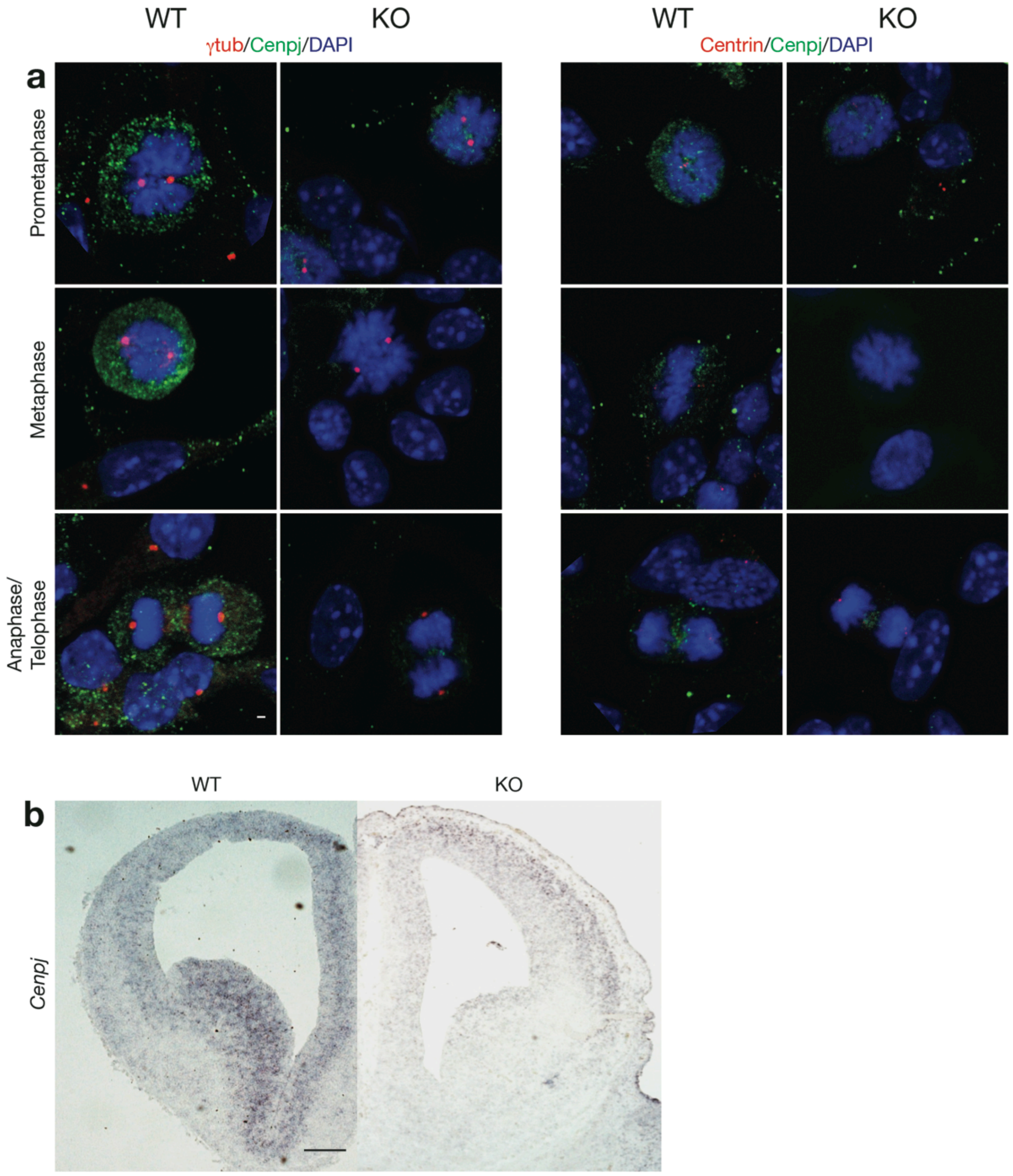

Supplementary Fig. 1 (Relative to Figure1) Ascl1 regulates *Cenpj* expression

a. Dissociated progenitors from E14.5 wild type and *Ascl1* null cortices were cultivated for 7 days. DAPI, γ-tubulin or centrin labeling were used to visualize single mitotic and interphase figures and their centrosomes and centrioles,

respectively. Cenpj is expressed throughout mitosis and interphase in cortical progenitors and downregulated in *Ascl1* mutant cells in all phases of the cell cycle. Scale bar= 1 μm.

b. In situ hybridization for *Cenpj* on coronal sections of at E14.5 telencephalon from wild type and *Ascl1* mutant mouse. Expression of *Cenpj* is stronger in the VZ of the ventral telencephalon and is reduced in the VZ of both dorsal and ventral telencephalon of the *Ascl1* mutant embryo. Scale bar=500μm.

Supplementary Figure 2

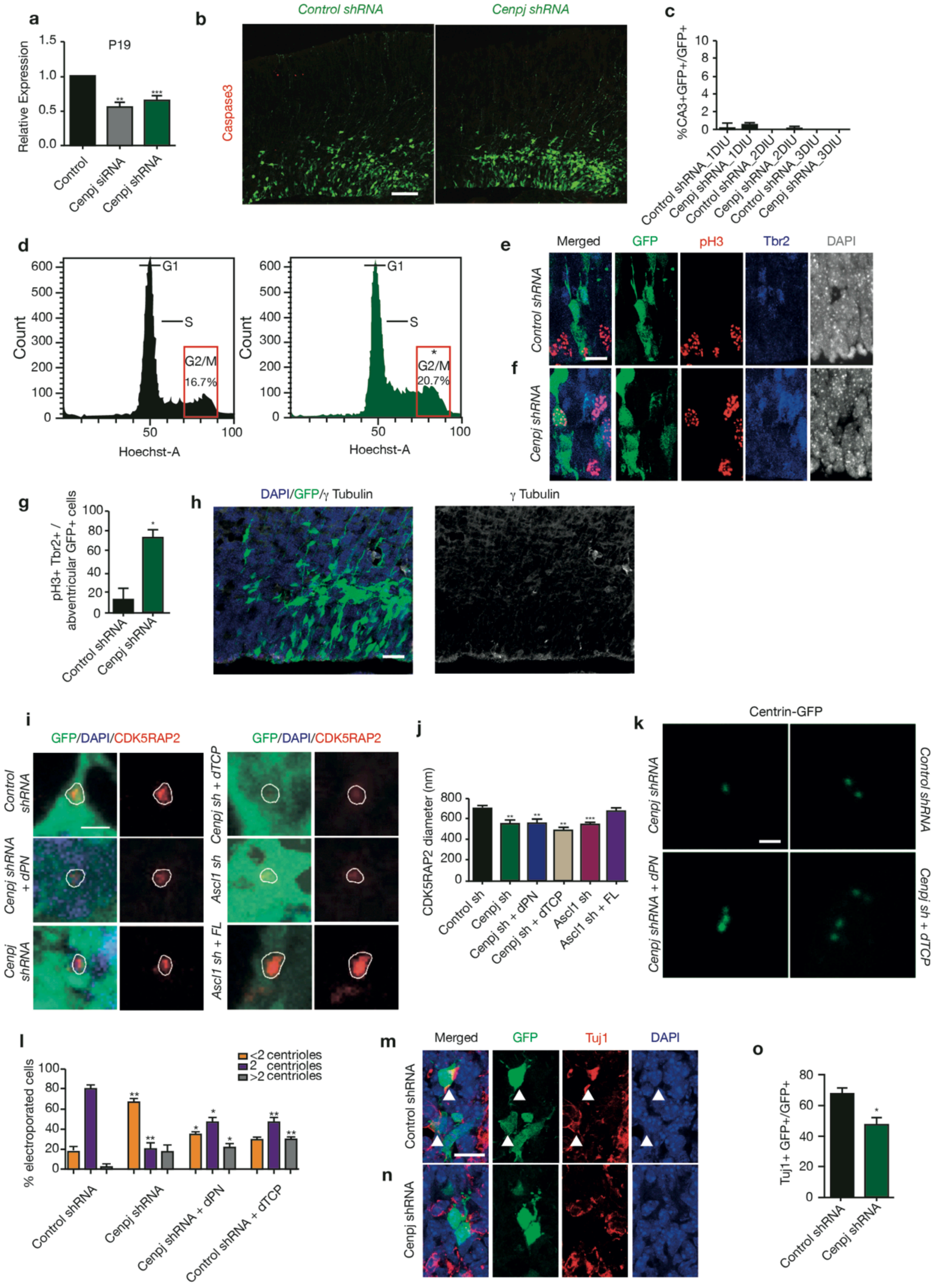

Supplementary Fig. 2 (Relative to Figure2) *Cenpj* is required for centrosome biogenesis.

a. The efficiency of *Cenpj* silencing was verified by transfecting shRNAs in P19 cells. 24 hours after transfection with control shRNA, *Cenpj* shRNA or *Cenpj*

siRNA, RT-qPCR was performed. Actin was used as a control to normalize levels of expression. Mean ± SEM, n=3, Student's t-test ** $p < 0.01$, ***$p < 0.001$

b,**c.** *In utero* electroporation of *Cenpj* shRNA in E14.5 cortices does not promote apoptotic cell death after one, two or three days *in utero*, as shown by the similar low fraction of electroporated GFP^+ cells (green) labelled with an antibody against activated caspase 3 (red) in control and *Cenpj* shRNA electroporated slices. Scale bar= 100μm. Data presented here as the mean ± SEM from at least six sections prepared from three embryos obtained from three litters for each condition in this analysis and subsequent quantifications, unless stated otherwise. Student's t-test non-significant.

d. Cell cycle analysis two days after electroporation of control or *Cenpj* shRNAs by flow cytometry showed an increase in the percentage of electroporated cells in G2/M phase when *Cenpj* was silenced. Percentages represent the mean of 3 experiments. Student's t-test * $p < 0.05$.

e-g. Quantification of $Tbr2^+pH3^+GFP^+$cells scattered in the VZ away from the apical surface one day after electroporation of the control or *Cenpj* shRNAs. *Cenpj* silencing resulted in an increased fraction of progenitors dividing away from the ventricular surface (Figure 2a and b) and 80% of these cells expressed the basal progenitor marker Tbr2. Scale bar= 10μm.

h. Immunostaining with γ-tubulin antibody to identify centrosomes aligned on the apical side of the VZ one day after *in utero* electroporation at E14.5. Scale bar=20μm.

i, **j** Analysis of the size of centrosomes labelled with cdk5rap2 in cortical neurons three days after co-electroporation of GFP with control shRNA*, Cenpj* shRNA*, Cenpj* shRNA together with a truncated *Cenpj* construct lacking the microtubule

destabilizing domain PN2-3 (dPN2-3) or *Cenpj* shRNA with a truncated *Cenpj* construct lacking the domain TCP (dTCP,). Measure of the centrosome diameter was done after a maximum projection of confocal images using ImageJ. Scale bar = 1 μm. Student's t-test ** $p < 0.01$, ***$p < 0.001$

k,l. Examples of centrin-GFP electroporated neurons displaying single, double or more than double centrioles (k). Number of centrioles quantification in cortical neurons three days after co-electroporation of centrin-GFP with a control shRNA, *Cenpj* shRNA, *Cenpj* shRNA and *dPN*, *Cenpj* shRNA and *dTCP*. Scale bar = 1 μm.

m,n. Immunostaining with Tuj1 antibody to identify neurons two days after *in utero* co-electroporation at E14.5 of GFP and control shRNA (m) or *Cenpj*-specific shRNA (n). Arrowheads point to cells with co-localized Tuj1 and GFP signals. Scale bar=20μm.

o. Quantification of the fraction of GFP^+ cells co-expressing Tuj1 in the whole cortical wall shows a reduction of neuronal production from *Cenpj*-depleted progenitors. Student's t-test * $p < 0.05$.

Supplementary Figure 3

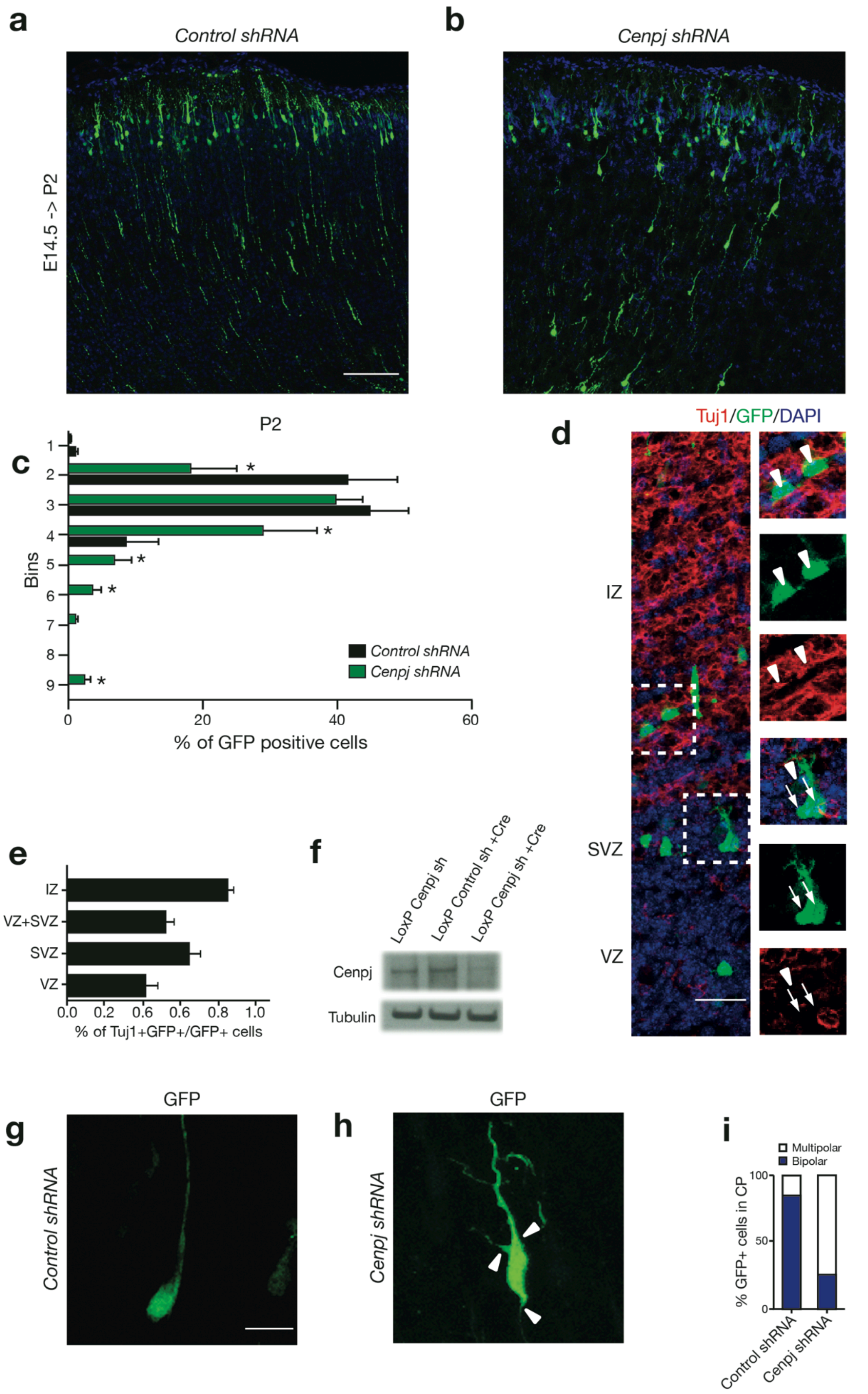

Supplementary Fig. 3 (Relative to Figure3) *Cenpj* is required for the

migration

a,b. Analysis of the position of post-migratory neurons in GFP-labelled cortical sections at P2 after *in utero* co-electroporation at E14.5 with GFP and control shRNA (a) or *Cenpj* shRNA (b). Scale bar= 100 μm.

c. Quantification of the position defect of neurons silenced for *Cenpj* by measuring the percentage of GFP^+ cells that have reached the different zones of the cortex. Electroporated cortices were subdivided into 9 bins. Cells silenced for *Cenpj* were mispositioned throughout the cerebral wall. Student's t-test * $p < 0.05$.

d. Double-staining for GFP (green) and the early neuronal marker Tuj1 (red) two days after in utero electroporation of *Control* shRNA in cortical section. Arrowheads point to co-stained GFP^+Tuj1^+ cells and arrows point to GFP^+Tuj1^- cells in the SVZ. Scale bar= 20 μm.

e. Quantification of the fraction of GFP^+ cells co-expressing Tuj1 in the different cortical compartments (VZ, SVZ and IZ). Mean ± SEM.

f. The efficiency of conditional *Cenpj* silencing was analysed by transfecting shRNAs in NS5 cells. 48 hours after transfection with pCALSL_*LoxP Cenpj* shRNA (c1), pCALSL_ *LoxP* control shRNA + pCAGScre (c2) or pCALSL_ *LoxP Cenpj* shRNA + pCAGScre (c3), proteins were extracted and Cenpj protein levels were analysed by western blot. Alpha tubulin was used as a loading control.

g,h. *Cenpj*-depleted neurons display multipolar processes three days after electroporation compared with bipolar control neurons analysed in the cortical plate. Scale bar: 10 μm.

i. Quantification of number of processes from the cell body, bipolar (two processes) or multipolar (more than two processes) performed three days after

electroporation. 3 embryos analysed for each condition; more than 200 cells analysed per condition.

Supplementary Figure 4

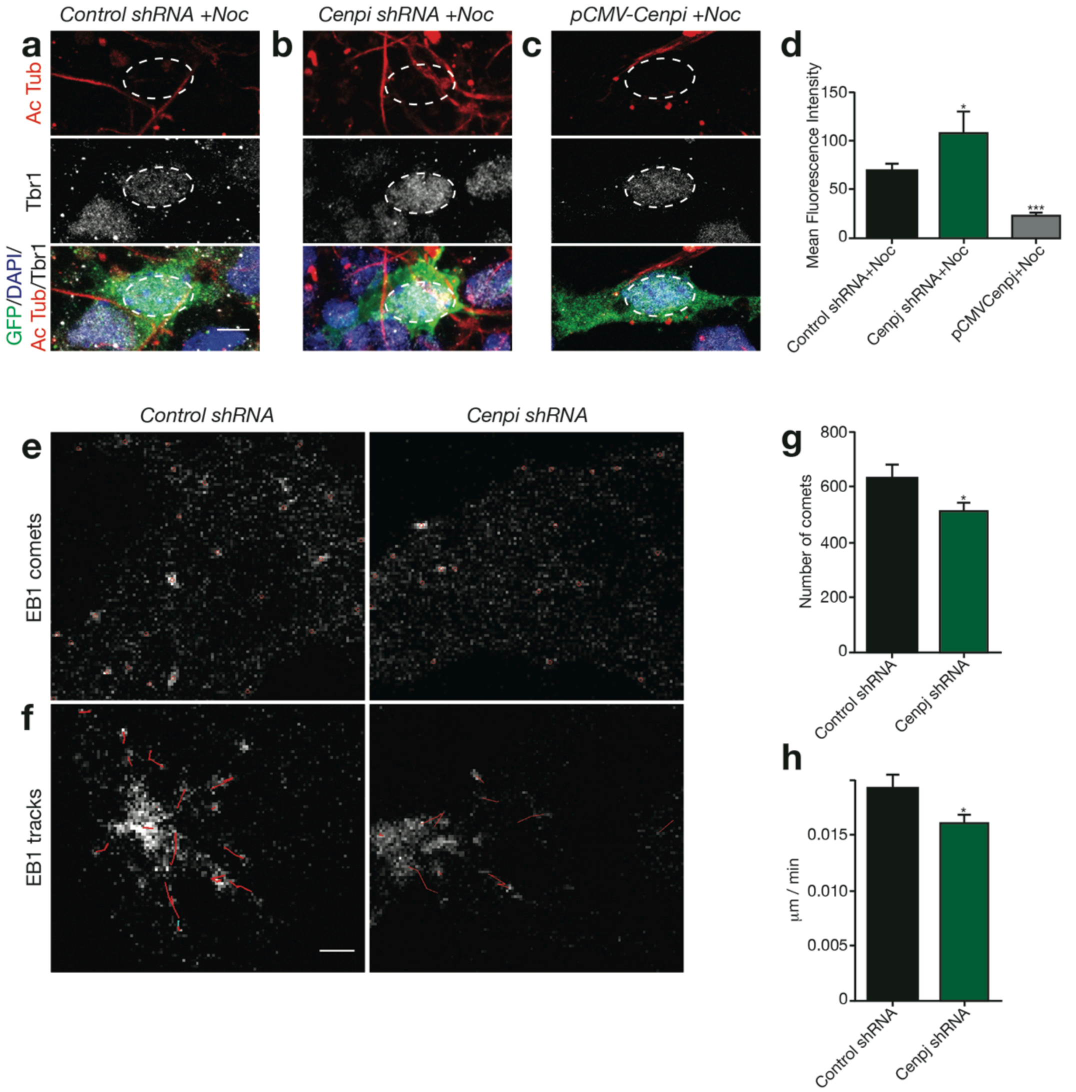

Supplementary Fig. 4 (Relative to Figure4) *Cenpj* is required for microtubule dynamics

a,b,c. Effect of nocodazole on stable acetylated microtubules in Tbr1^{+} neurons cultured *in vitro* for three days after *ex vivo* electroporation of GFP and control,

Cenpj shRNAs or *Cenpj* overexpression vector. White line shows DAPI limits. Scale bar: 3 μm.

d. Quantification of acetylated tubulin labelling co-localized with the nucleus in nocodazole-treated cultures*. Cenpj* overexpression results in a reduction of acetylated tubulin staining and therefore in a decrease of stable microtubules in the microtubule cage Analysis of 3 independent cultures; control shRNA, n=40 cells; *Cenpj* shRNA, n=42. *Cenpj* overexpression, n=48. Student's t-test *$p < 0.05$, ***$p < 0.001$.

e-h. Maximum projections of time-lapse recordings of End-Binding protein 1 (EB1)-GFP after 2 DIV. EB1-GFP comets (e) and the tracks (f) are indicated in red. White line shows cell membrane limits. Quantification of the number of comets (g) and the speed of tracks measured in μm/min (h). Mean of 3 independent cultures, at least 9 movies per condition. Student's t-test * $p < 0.05$. Scale bar: 1 μm.

Supplementary Figure 5

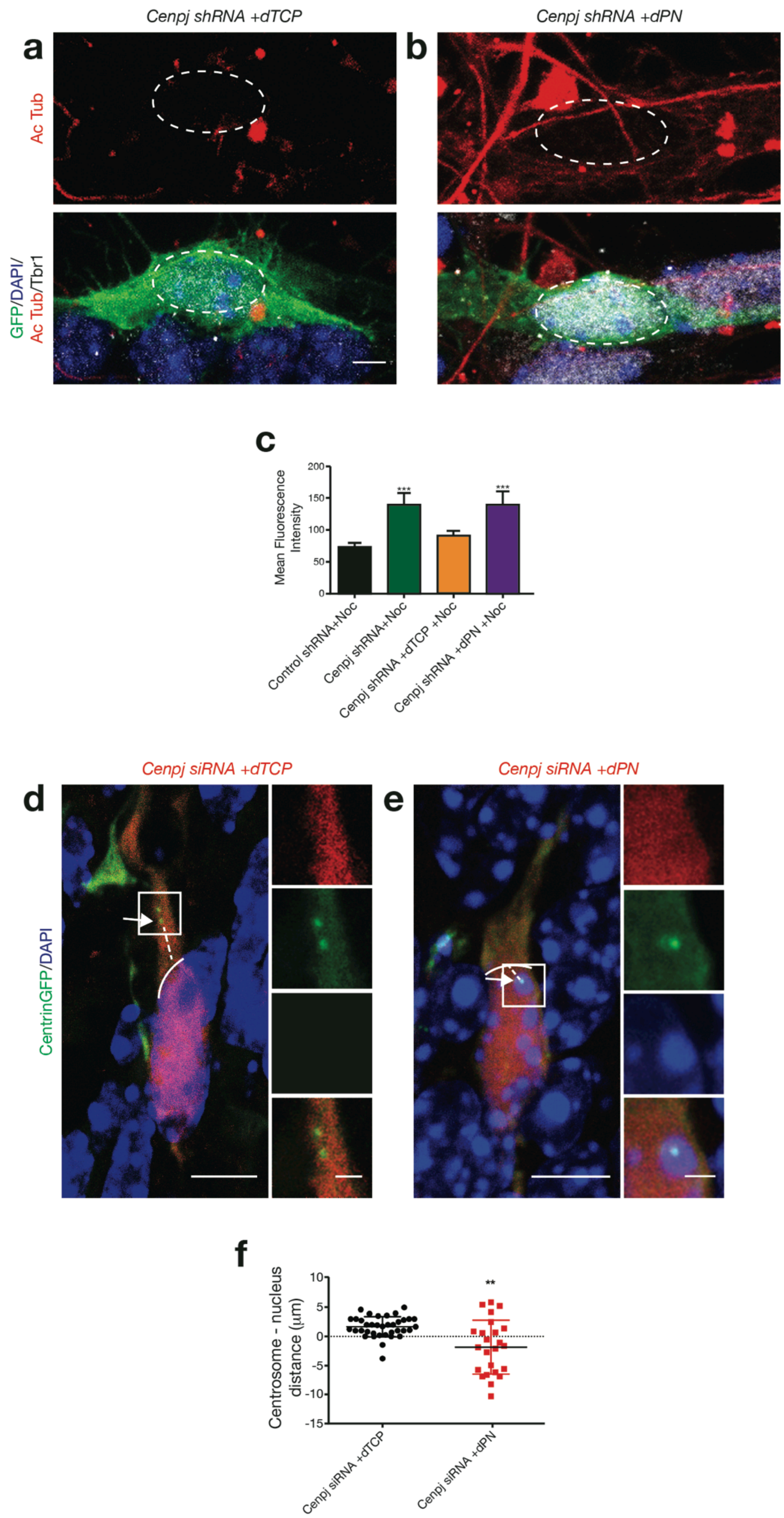

Supplementary Fig. 5 (Relative to Figure5) The PN2-3 protein domain is required for microtubule regulation

a,b,c. Nocodazole treatment of cultured neurons three days after electroporation. *Cenpj* shRNA *+ dTCP* rescued the labelling of acetylated tubulin over the nucleus to a level comparable to that of control shRNA-electroporated cells. In contrast, *Cenpj* shRNA *+ dPN* maintained the high level of acetylated tubulin found in *Cenpj* shRNA-electroporated cells. Scale bar= 3 μm.

d,e. Analysis of the distance between the centrosome and the nucleus in migrating cortical neurons three days after *in utero* co-electroporation at E14.5 of *Cenpj* shRNAs +dTCP (d) or dPN (e) together with pClG2-Centrin2-Venus to label the centrosome and pCMV-RFP to mark electroporated cells. The nuclei are labelled with DAPI. Insets to the right show higher magnification of the boxed areas with separate colour channels. Arrows point to the centrosome, dashed line represents the distance between the centrosome and the tip of the nucleus, marked by a full line. Scale bar: 3 μm (main) and 1μm (boxed insets).

f. Quantification of the distance between the centrosome and the nucleus in migrating cortical neurons. Negative values correspond to centrosomes located below the tip of the nucleus. dPN did not rescue the centrosome-nucleus coupling defect from *Cenpj*-depleted neurons. 3 embryos analysed for each condition; *Cenpj* shRNA + dTCP, n=37 cells; *Cenpj* shRNA, + dPN n=24. Student's t-test ** $p < 0.01$.

Supplementary Figure 6

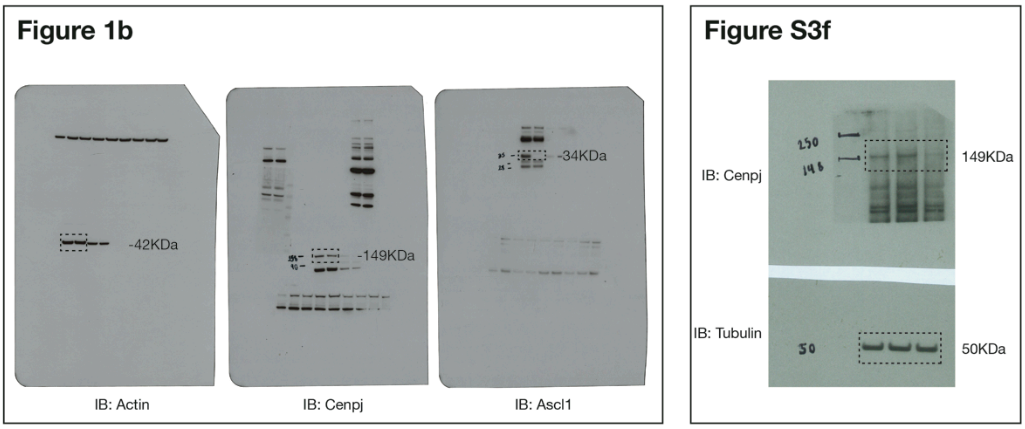

Supplementary Fig. 6 Full blots

Full blots from Figure 1b and supplementary figure 3f. Dashed black rectangles represent cropped areas displayed in Figures.

SUPPLEMENTARY METHODS:

Flow cytometric analysis

After *ex vivo* electroporation, cortical cells were dissociated mechanically and enzymatically with Accutase (Sigma) and plated into proliferation medium described above at a density of 65,000 cells/cm^2. After two days *in vitro*, cells were dissociated and incubated with Hoechst 33342 (10µg/ml, Molecular Probes) at 37^0C for 45 minutes. Cells were analyzed on a BD FACS Calibur. Cell cycle phase distributions were calculated with Watson's pragmatic model within the Flowjo software package (Tree star Inc.).

Immunostaining

After washing in PBS, sections were treated with PBS - 0.1% TritonX100 - 10% serum for 30 minutes. They were then incubated overnight at 4°C with the following primary antibodies diluted in the blocking buffer: rabbit anti-beta III

Tubulin (1:100, Abcam); rabbit anti-Cdk5rap2 (1:100, Milipore); goat anti-pH3 (1:50, Santa Cruz); rabbit anti-TBR1 (1:100, Abcam) and rabbit anti-TBR2 (1:100, Abcam).

Live imaging and analysis

Analysis of microtubule dynamic was performed using the plus-end growth marker EB1. Two days after *ex vivo* electroporation, EB1-GFP images were captured at 37^{0}C from live cultured neurons using inverted Leica SP5 confocal microscope. A 100x 1.46 N.A. oil immersion objective was used giving pixel size of 100nm/pixel. Laser line used was 488 nm. Comets numbers and tracks speed was calculated using a ClusterTrack software package available online at http://lccb.hms.harvard.edu/software.html with Matlab (MathWorks). Images were acquired at 1 frame per second. Total recording time: 1 min 20 seconds. 5X speed up.
